# A Spatial Panel Data Analysis of Economic Growth, Urbanization, and NO_x_ Emissions in China

**DOI:** 10.3390/ijerph15040725

**Published:** 2018-04-11

**Authors:** Xiangyu Ge, Zhimin Zhou, Yanli Zhou, Xinyue Ye, Songlin Liu

**Affiliations:** 1 School of Statistics and Mathematics, Zhongnan University of Economics and Law, Wuhan 430073, China; xiangyu_ge@hotmail.com; 2 School of Finance, Zhongnan University of Economics and Law, Wuhan 430073, China; 3 Computational Social Science Laboratory, Kent State University, Kent, OH 44240, USA; xye5@kent.edu; 4 Faculty of Mathematics and Statistics, Hubei University, Wuhan 430062, China; roger007@sina.com

**Keywords:** nitrogen oxides emissions, urbanization, sustainable development, EKC, spatial effects

## Abstract

Is nitrogen oxides emissions spatially correlated in a Chinese context? What is the relationship between nitrogen oxides emission levels and fast-growing economy/urbanization? More importantly, what environmental preservation and economic developing policies should China’s central and local governments take to mitigate the overall nitrogen oxides emissions and prevent severe air pollution at the provincial level in specific locations and their neighboring areas? The present study aims to tackle these issues. This is the first research that simultaneously studies the nexus between nitrogen oxides emissions and economic development/urbanization, with the application of a spatial panel data technique. Our empirical findings suggest that spatial dependence of nitrogen oxides emissions distribution exists at the provincial level. Through the investigation of the existence of an environmental Kuznets curve (EKC) embedded within the Stochastic Impacts by Regression on Population, Affluence, and Technology (STIRPAT) framework, we conclude something interesting: an inverse N-shaped EKC describes both the income-nitrogen oxides nexus and the urbanization-nitrogen oxides nexus. Some well-directed policy advice is provided to reduce nitrogen oxides in the future. Moreover, these results contribute to the literature on development and pollution.

## 1. Introduction

China’s economy has developed at an incredibly fast pace for decades and received broad attention. By 2010, China’s gross domestic product (GDP) reached 5.8 trillion dollars, making China the world’s second largest economy (NBSC 2011b). The country is turning from an agricultural one to a modernized one [1], with more than half of the population living in urban areas (NBSC 2011b). In the meantime, the rapid economic growth and urbanization came with a rocketing consumption of resources and a soaring emission of air pollutants, with nitrogen oxides (NO_x_) being the fastest accelerating air pollutant in China in the last two decades [2,3]. NO_x_ is an important air pollutant because it contributes to the formation of photochemical smog, which can have significant impacts on human health. The main adverse effects of NO_x_ to public health is that it causes respiratory diseases. Chronic exposure to NO_x_ under ultraviolet radiation can cause respiration symptoms in people with asthma, and bronchial symptoms (especially in children) and airway inflammation in healthy people. In addition, NO_x_ is the main source of nitrate aerosol (the important component of particulate matter (PM) 2.5) in China [3,4,5].

The literature on the relationship between economic development and environmental quality is extensive in the field of environmental economics. The present empirical study relies on the Environmental Kuznets Curve (EKC) hypothesis carried out by Grossman and Krueger [6] because its expanded form has the potential to be a policy tool for sustainable development [7]. Some researchers have examined NO_x_ EKC through cross-sectional data [8,9] and panel data [10,11]. Most researchers applied country-level data to explore sulfur dioxide (SO_2_) EKC, while only a few studies used prefecture-level data in some specific countries.

So far, few studies have explored the relationship between nitrogen oxides emissions and socioeconomic factors through quantitative empirical approaches in the context of China, even though such empirical analyses are sorely urgent, because they can shed some new light on the driving forces and precise regularities of pollutants emission levels, and the estimated parameters and functions could be very helpful for policymakers to implement suitable policies for emission reduction. Brajer, et al. [12] carried out the solely related study through panel data. Although panel data have relatively more information and greater degrees of freedom than cross-sectional data, spatial dependence is a problem in many panel datasets when the individuals are not sampled at random. In reality, an observation in a cross-sectional sample is always related to some other observations in the same sample [13]. Anselin and Griffith [14] illustrated this phenomenon as the existence of a relationship between what occurs at one spot in space and what occurs somewhere else. However, such a relation certainly violates the pre-assumption for standard regression analysis: the sampled observations should generally be independent of each other.

Moreover, spatial effects are critical factors in assessing the impact of the economic development on environmental conditions [15,16]. The spatial correlation of data is an inherent characteristic in many environmental subjects. The spread of waste effluents, the diffusion of air pollutants, and the invasion of new species all might bring about spatial autocorrelation that hinders statistical analysis [17]. According to some recent studies on air pollution, China’s air pollutants show a spatially correlated pattern [18,19,20,21]. For the establishment of EKC, regional samples (towns, cities, or provinces) located nearby may interact because of spillovers of economic factors and pollutant emission regulations [22].

Quite a few air pollution studies did find some evidence supporting an EKC hypothesis in China; however, the shape is mixed and different depending on indices as well as sample characters. Brajer, Mead, and Xiao [12] found evidence supporting an N-shaped sulfur dioxide (SO_2_) EKC. They investigated the existence of an SO_2_ EKC through a Chinese annual panel dataset consisted of 128 cities from 1990 to 2004 and obtained the classical inverted U-shaped relationship and an N-shaped EKC for SO_2_ emission. However, Song et al. [23] criticized that previous researchers failed to consider the potentially non-stationary time series and panel data. Thus, they applied a panel cointegration technic to improve the non-stationarity issue and obtained an inverse-U shape EKCs for exhaust gas, wastewater, as well as solid wastes. Diao et al. [24] applied 11-year data (1995–2005) to search for EKC for six kinds of pollutants (four of them are air pollutants) in Zhejiang province. They obtained an inversely U-shaped trajectory for the emission of Industrial exhaust gas, smoke, and ash. They likewise obtained a positive linear nexus for SO_2_ emission and income. Nevertheless, such a linear relation did not necessarily eliminate an EKC, since this province might just have been going through the first upward phase of the inverted-U shape.

Some recent studies adopted advanced econometric tools in China’s economic environment analysis. On the basis of the examination of a provincial panel in the period 1990–2012, Wang et al. [25] obtained an inverse U-shaped curve for the relationship between income and SO_2_ emission and a positive linear relation between urbanization and SO_2_ emission by semi-parametric regression for the first time. Zheng, Yu, Wang, and Deng [20], and Kang et al. [26] applied a spatial panel data approach in order to control for variables’ spillover effects in their analysis of the connection between carbon dioxide (CO_2_) emission and economic growth. They both found empirical evidence for an inverse-N shaped curve. Hao and Liu [18] used spatial econometric tools to examine China’s current severe air pollution, i.e., particulate matter (PM) 2.5 from cross-sectional data of 73 Chinese cities in 2013; the outcome suggested a standard inverse-U shaped EKC for the relation between PM 2.5 concentration and income.

Some supporters of modernization theories argue that a relationship of EKC exists between environmental impact and urbanization rather than economic development [27,28,29]. However, the important issue of urbanization’s impacts on nitrogen oxides emissions in China is left undiscussed in the past literature.

Therefore, the objectives of our study are to systematically estimate the impacts of income and urbanization (hereinafter referred to as “income/urbanization”) on nitrogen oxides emissions. Since the nitrogen oxides emissions data and other socioeconomic indicators are all sampled at the provincial level of contiguity, we naturally applied the spatial panel model as the grounding technique in the following empirical analysis. We begin with a brief introduction to provide the theoretical basis and methodology for the following empirical analysis. Then, this paper proceeds with global Moran’s I test and conventional regression to get diagnostics for the description of spatial dependence. In addition, we investigate the relation between the driving forces and the dependent variable thoroughly with the spatial panel tool. The outcomes are presented and discussed; besides, we offer some policy advice at the end of this study.

This research mainly contributes to the current literature in the following aspects. First, we investigate the relation between nitrogen oxides emissions and economic development in the context of China, especially the relationship between nitrogen oxides emissions and economic growth/urbanization. As far as we know, this is the first empirical estimation of the impact of socioeconomic influential factors on nitrogen oxides emissions in China with the EKC and the STIRPAT (Stochastic Impacts by Regression on Population, Affluence, and Technology) model. Second, the spatial panel data tools are applied for the empirical analysis, so that the spatial dependence of nitrogen oxides emissions can be taken into account and the biased estimators caused by omitting the spatial effects can be avoided. So far, no prior quantitative analysis of the nexus between socioeconomic factors and nitrogen oxides emissions has utilized spatial econometric tools. Third, we rectify the previous way of calculating the turning points used in those EKC studies that applied a spatial econometric approach.

## 2. Theoretical Framework and Methodology

### 2.1. Environmental Kuznets Curve Hypothesis

EKC is originally an empirical hypothesis that characterizes an inversely U-shaped curve for the relationship between economic development and environmental quality. Various indices of environmental quality degenerate with economic growth. After reaching a threshold, the environment deterioration starts to decrease [6]. Development may promote environmental quality as a result of economies of scale from pollution reduction, technological upgrade, industrial structure escalation, and public’s demand for a clean environment [22]. Generally, the considered model for the EKC is a polynomial function type as follows:

(1) $$Y=\alpha +\beta_{1}X+\beta_{2}X^{2}+\beta_{3}X^{3}+\beta_{4}Z+\varepsilon$$

Here, *Y* represents the indices of environmental degradation, while X refers to the economic development level, usually measured by per capita GDP (Gross Domestic Product), and Z includes other influential factors for the environment. The polynomial function form of EKC offers to us an adequate tool to estimate the nonlinear relationship (if it exists) between economic growth/urbanization and pollutants emission.

### 2.2. Stirpat Model

We use the STIRPAT model as our theoretical foundation to test the existence of an EKC for nitrogen oxides emissions related to affluence. Ehrlich and Holdren [30] first proposed the concept of IPAT (Impact, Population, Affluence, and Technology), a model describing the impacts of population, affluence and technology on the environment, while Commoner et al. [31] applied it by algebraic formulation to data analysis. The IPAT identity is concise and well ecologically grounded. However, the IPAT model is only an overly simplified function form and just indicates that the impact of human activities on the environment can fully be differentiated into population, affluence, and technology effects. Thus, the IPAT model cannot estimate to what extent a specific factor affects the environment in such a framework, not to mention test any hypothesis. Another limitation is that it assumes that only fixed proportionality changes happen between effects and factors. Therefore, Dietz and Rosa [32] derived a stochastic version of IPAT, known as STIRPAT and later refined by York et al. [33], expressed by the equation:

(2) $$I_{i}=\alpha_{i}P_{i}{}^{b}A_{i}{}^{c}T_{i}{}^{d}\varepsilon_{i}$$

Here, I represents the environmental impact, P,A, and T indicate human activities, i.e., respectively, population, affluence (per capita), and technological influences (per unit of economic activity); α,b,c,d are coefficients to be estimated; ε is the error (the proportionality of IPAT pre-assume α=b=c=d=ε=1). The subscript *i* refers to the *i*th region, and as indicated by *i*, quantities of , and vary across observations. Its regression form for estimation and hypothesis test is obtained by logarithmic transformation of the variables in Equation (1). In this case, the coefficients b,c, and d stand for the Ecological Elasticity (EE) which measures the sensitivity of environmental impacts to a change occurring in a driving force. It is defined as the proportion of change in environmental impacts due to its significant determinants. Since it is highly flexible to various functional forms, a quadratic or higher term of affluence can enter the STIRPAT equation [33]. Therefore, we applied an augmented STIRPAT for our study purpose:

(3) $$\ln I=\alpha +b_{1}\ln A+b_{2}{\left(\ln A\right)}^{2}+b_{3}{\left(\ln A\right)}^{3}+c\ln P+d\ln T+e$$

(4) $$\ln I=\alpha +b_{1}\ln A+b_{2}{\left(\ln A\right)}^{2}+c\ln P+d\ln T+e$$

(5) lnI=α+blnA+clnP+dlnT+e

According to our study purpose and to modernization theories [27,29], per capita GDP and the percentage of urban population (to reflect the modernization level) are utilized as the proxies of affluence; energy intensity is defined as the indicator of technology impacts/damages [28]. Environmental impact refers to the amount of nitrogen oxides emissions.

### 2.3. Spatial Panel Data Model

Before the statistical inference for spatial model specifications is carried out, we make a brief introduction to the models. Three currently prevailing spatial panel models were considered: the spatial Durbin model (SDM), the spatial lag model (SLM), and the spatial error model (SEM). The SDM model can be written in matrix form as:

(6) $$Y=\delta \left(I_{T}⊗W_{N}\right)Y+X\beta +\gamma \left(I_{T}⊗W_{N}\right)X+\left(\tau_{T}⊗I_{N}\right)\mu +\left(I_{T}⊗\tau_{N}\right)\eta +u,\;u∼N\left(0,\sigma^{2}I_{NT}\right)$$

The SLM model can be written as:

(7) $$Y=\delta \left(I_{T}⊗W_{N}\right)Y+X\beta +\left(\tau_{T}⊗I_{N}\right)\mu +\left(I_{T}⊗\tau_{N}\right)\eta +u,\;u∼N\left(0,\sigma^{2}I_{NT}\right)$$

The SEM model can be written as:

(8) $$\begin{array}{l} Y=X\beta +\left(\tau_{T}⊗I_{N}\right)\mu +\left(\tau_{N}⊗I_{T}\right)\eta +u\\ u=\rho \left(I_{T}⊗W_{N}\right)u+\upsilon \\ \upsilon ∼N\left(0,\sigma^{2}I_{NT}\right) \end{array}$$

In this study, the dependent variable *Y* is substantively an *NT* × 1 vector of nitrogen oxides emissions amount at China’s provincial level, and *X* is an *NK* × *K* matrix composed of independent variables (also known as explanatory variables) in Equations (3)–(5); *μ* controls for the unknown individual effects or heterogeneities (each individual province’s features that might affect emissions level but never changes over time. For example, the geographical distribution of provinces) of the 30 provinces in the study, since omission can lead to biased estimates, while η controls for the time effects, i.e., the constant whole trend of nitrogen oxides emissions levels; *δ* is the spatial autocorrelation and *ρ* is the spatial autocorrelation that exists in the error term *u* (the error term in regression analysis is usually assumed as a random variable of normal distribution); υ is the random term existing in the spatially correlated *u*. Both *δ* and *ρ* reflect the strength of the dependent variable’s spatial autocorrelation. Each parameter indicates the responsiveness of the dependent variable to a change in the independent variables; *γ* is the coefficient showing the spillover effects of the independent variables on the dependent variable; $\tau_{N}$ is a column vector of all vectors in the length of *N*, while $\tau_{T}$ represents a column vector of all vectors in the length of *T*; $I_{N}$ and $I_{T}$ are *N* × *N* and *T* × *T* dimension identity matrixes, respectively.

$W_{N}$ is the *N* × *N* weight matrix, in which the elements represent the contiguity of provinces. The element on the *i*th row and *j*th column equals 1 if the *i*th province and *j*th province have a mutual border, otherwise, it equals 0. As a routine, $W_{N}$ is always row-normalized in spatial econometric analysis, and the elements in normalized $W_{N}$ are between 0 and 1 [34]. To capture the spatial autocorrelation and spillover effects in the model with panel datasets, the weight matrix is constructed as $W_{NT}=I_{T}⊗W_{N}$, where ⊗ indicates the Kronecker product.

To show the necessity and the advantage of applying the spatial panel model rather than the traditional panel data methodology, we report the results of the non-spatial panel data model as well. The classical Lagrange Multiplier (LM) and robust LM tests come along with these results because the LM tests can offer inferences for adopting the SLM or SEM. After the LM tests, the estimated results of the SDM, SLM, and SEM are analyzed by the Wald as well as the LR statistics. If the null hypothesis of the Wald test (*H*_0_: γ=0) cannot be rejected, then the SDM can be simplified to the SLM, and if null hypothesis of the LR test (*H*_0_: γ+δβ=0) cannot be rejected, then the SDM can be simplified to the SEM [35]. If both null hypotheses are rejected, then the SDM remains reasonable. On the other hand, if *H*_0_: γ=0 holds true and the LM/LM robust tests of the SLM rejects its null hypothesis *H_0_*: no spatial lag effects, then the SLM can be considered. Similarly, if *H*_0_: γ+δβ=0 is not rejected, while the LM/LM robust test of the SEM rejects its null hypothesis *H*_0_: no spatial lag effects in error term, then the SEM should be adopted. Otherwise, the SDM is still preferred because of its relatively better flexibility. For example, if the Wald/LR tests suggests a model different from the one suggested by the LM tests/robust LM tests, the SDM should still be preferred [34]. Lastly, the Hausman test is to be conducted to determine whether fixed individual effects or random individual effects should be adopted.

## 3. Data and Variables

This paper investigates the nexus of income/urbanization to nitrogen oxides emissions through a balanced panel dataset of 30 provinces in China, spanning from 2010 to 2015 (data of Tibet autonomous province, Taiwan province, Hong Kong, and Macau special administrative regions were not available.). The data on 2010–2015 nitrogen oxides emissions, income (per capita GDP), urban population, and total population all originate from the National Bureau of Statistics of China. The nitrogen oxides emissions amount in 2010 was obtained from the webpage of the Ministry of Environmental Protection of the People’s Republic of China. Energy consumption (kg of coal equivalent) data were collected from the China Energy Statistical Yearbook. The per capita GDP data was converted into the 2003 constant price. Table 1 lists all the definitions and descriptive statistics of the variables. All variables were processed with natural logarithm transformation. Logarithm transformation can diminish the potential estimation bias caused by the large scope of data values.

## 4. Empirical Results and Discussion

### 4.1. Spatial Distribution of Nitrogen Oxides in China

We first explore the possible existence of a nitrogen oxides emissions’ spatial autocorrelation during the data interval. Global Moran’s I statistics is a widely applied index for spatial autocorrelation detection. It reflects the spatial autocorrelation of whole areas of interest with a solo value and depends on the spatial weight matrix that shows the geographic relationship among samples in adjacent regions. In that way, it assesses the observations’ distribution pattern: random, clustered, or dispersed. Here, the Equation (9) is the formula of Global Moran’s I:

(9) $$I=\frac{\sum_{i=1}^{N}\sum_{i\neq j}^{N}w_{ij}\left(x_{i}-\bar{x}\right)\left(x_{j}-\bar{x}\right)}{{\left(\sum_{i}x_{i}-\bar{x}\right)}^{2}\sum_{i=1}^{N}\sum_{i\neq j}^{N}w_{ij}}$$

where $\bar{x}=\frac{1}{N}\sum_{i=1}^{N}x_{i}$; $w_{ij}$ is the element on the *i*th row and *j*th column of the spatial weight matrix W, and N is 30 in our study (the number of provinces); x is the indicator of interest. In this research, W is characterized by the commonly accepted specification, i.e., fist-order Rook Adjacency (China’s spatial weight matrix of rook contiguity is the same as the matrix of queen contiguity). The significance of Global Moran’s I is usually testified by the Z-score (the comparison of Moran’s I and its expectation). The calculation of *Z* statistics was done through Equations (10)–(12) ($w_{i}$ and $w_{j}$ are the sum of the *i*th row and *j*th column of the spatial weight matrix W, respectively). All the empirical results in this study are generated through Matlab and ArcGIS. Table 2 illustrates the Global Moran’s *I* of nitrogen oxides emissions from 2010 to 2015.

(10) $$Z=\frac{I-E(I)}{\sqrt{Var(I)}}$$

(11) $$E(I)=-\frac{1}{n-1}$$

(12) $$Var(I)=\frac{n^{2}w_{1}+nw_{2}+3w_{0}{}^{2}}{w_{0}^{2}(n^{2}-1)}-E^{2}(I)$$

$$w_{0}=\sum_{i=1}^{n}\sum_{j=1}^{n}w_{ij}\;w_{1}=\frac{1}{2}\sum_{i=1}^{n}\sum_{j=1}^{n}{\left(w_{ij}+w_{ji}\right)}^{2}\;w_{2}=\sum_{i=1}^{n}\sum_{j=1}^{n}{\left(w_{i}+w_{j}\right)}^{2}$$

The statistical significances of the Moran’s I are presented by their Z score and corresponding *p* values. As shown above, the Z scores and *p* values clearly state that the spatial autocorrelation effects in nitrogen oxides emissions are significant at a 5% level over six years. The positive Moran’s *I* values indicates that the areas with high nitrogen oxides emissions (provinces in the high-high groups) tend to locate together, like the low emission areas (provinces in the low-low groups). During 2010–2015, the decreased Global Moran’s *I* suggests a declining tendency of the agglomeration on nitrogen oxides emissions in China.

**Figure 1:**
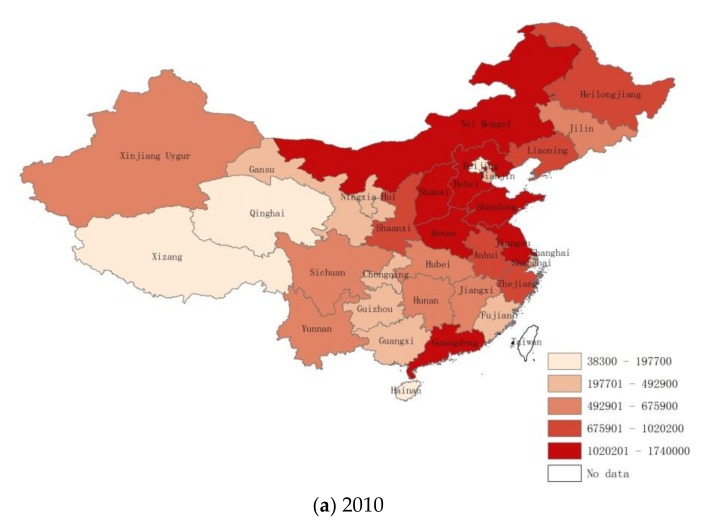

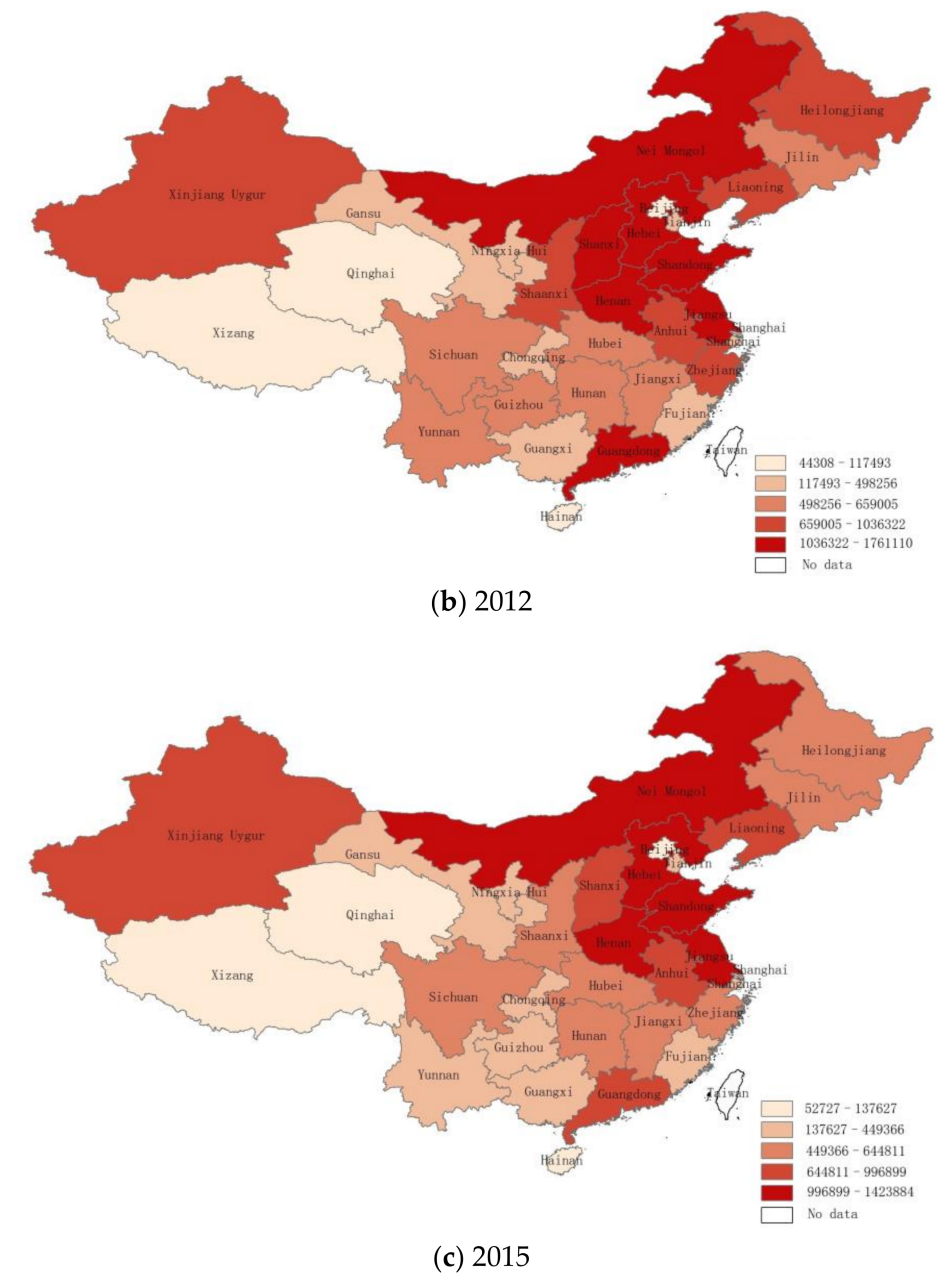
China’s NO_x_ emissions distribution in (**a**) 2010, (**b**) 2012, and (**c**) 2015, respectively (Units: tons).

In order to visualize and depict the spatial clustering pattern of nitrogen oxides emissions at a provincial level more intuitively, Figure 1 demonstrates the emission distribution in provinces in 2010, 2012, and 2015.

As shown below, the high-high (HH) cluster is mostly located in the eastern and northern regions of China and can be classified into two categories. One category is located in areas with dense population, high urbanization level, and developed economy, mostly in the eastern part of China (Henan, Shandong, Jiangsu, Shanghai, Zhejiang, etc.). The other category located in areas that heavily rely on heavy and mining industries, especially in the northeastern regions (Jilin, Liaoning, Hebei, Shanxi provinces, etc.). The low-low (LL) cluster regions of nitrogen oxides emissions are mainly located in undeveloped areas and areas of low population density, particularly in the north, middle, and south parts of China (Gansu, Ningxia, Shanxi, Chongqing, Guizhou, Guangxi, Yunnan, etc.).

To sum up, the geographical agglomeration of nitrogen oxides emissions is statistically significant during our study period, and the discharge of pollutants seemingly correlate with economic development and population effects. Specifically, wealthy provinces/cities with a large population generally have a higher emissions levels. This phenomenon corresponds to the STIRPAT model’s theory. In the next sub-section, we will explore the specific quantitative relationship between nitrogen oxides emissions and their driving forces.

### 4.2. Econometric Results and Analysis

#### 4.2.1. Non-spatial Panel Data Results

To determine the most appropriate model specification, this part firstly applies the non-spatial panel model to calculate classical LM and robust LM statistics for model specification (SLM or SEM). If the (robust) LM tests reject the non-spatial models, we will further determine which spatial panel model is the most appropriate one by the procedure discussed in Section 2.2: the estimated results for the SDM will testify if it can be simplified to the SLM or SEM. Once the most appropriate model is specified, we will estimate the driving forces’ direct and indirect marginal influences (if they exist) on pollutant’s emission, and then explain and discuss the results obtained.

Table 3 depicts the statistical results of regression models that control for both spatial fixed and time-fixed effects (two-way fixed effects) in two fields: GDP–nitrogen oxides and urbanization (URB)–nitrogen oxides. In each field, the estimated results of three different model specifications (M1–M3 indicating cubic, quadratic, and first terms of affluence models, respectively) are shown in three separated columns. The likelihood ratio (LR) test is conducted to verify the incorporation of two-way fixed effects against the incorporation of either spatial fixed or time-fixed effects. As we can see, the LR test results in Table 3 overwhelmingly reject the null hypothesis of spatial fixed effects as well as that of time period-fixed effects. Therefore, the two-way fixed effects are preferred over the spatial/time fixed effects in both GDP–nitrogen oxides and URB–nitrogen oxides models.

The LM tests significantly reject the null hypothesis of no spatially lagged dependent variable (nitrogen oxides) and no spatially auto-correlated error for the GDP–nitrogen oxides and Urbanization–nitrogen oxides models; however, the robust LM tests do not. This gives very ambiguous evidence for the validity of the spatial model. As mentioned in Section 2.2, for the final determination of which spatial panel model fits our data best, we need to consider the LR and Wald tests results. We illustrate these tests results in the following section.

#### 4.2.2. Spatial Panel Data Results

We will now turn to the spatial econometric analysis. Table 4 and Table 5 report the estimated results of the SDM model that controls for both spatial and time effects. Two fields (fixed effects estimates and random effects estimates) with triple columns contain these results in each table. In Table 4, the three columns in each field list and compare the results of three model specifications: the model with a cubic term of GDP (M1), the model with a quadratic term of GPD (M2), and the model with a linear term of GDP (M3). In a similar way, Table 5 compares the estimated results of the three models incorporating urbanization’s cubic, quadratic, and linear terms.

As shown in Table 4 and Table 5, Hausman tests (against fixed effects) under the three model specifications all reject the null hypothesis: the unobserved individual effects in the provinces are not correlated with the independent variables in the models. Thus, we only focus on the results of the GDP–nitrogen oxides and urbanization–nitrogen oxides models with two-way fixed effects in the following discussion.

When including two-way fixed effects in M1 and M2, all the Chi-square statistics of all LR and Wald tests of the GDP–nitrogen oxides and urbanization–nitrogen oxides models reject both hypotheses, *H*_0_: γ=0 and *H*_0_: γ+δβ=0. In other words: the SDM cannot be simplified to either the SLM or the SEM if one of the polynomial models is adopted. On the other hand, the Wald and LR tests in M3 (Table 4 and Table 5) do not reject their null hypothesis.

It is noteworthy that the coefficients’ estimates in the non-spatial model indicate the marginal effects of the driving forces (population and energy intensity) on the dependent variable (nitrogen oxides emissions), whereas the parameters’ estimates in the SDM or SLM do not. Instead, the independent variables’ direct and indirect (spillover) effects on the SDM need to be calculated by Equation (13), and the estimate results are reported in Table 6 and Table 7. Equation (13) is derived from Equation (14), and Equation (14) from Equation (6). The reciprocal term ${\left(I-\delta W\right)}^{-1}$ is calculated by Equation (15). All the parameters that need to be brought into Equations (13) and (15) are already estimated and reported in Table 4 and Table 5. The diagonal elements of the partial derivatives matrix in Equation (13) indicates the direct effects (elasticity) of the kth explanatory variable, and all the off-diagonal elements refer to its spillover effects. Consequently, if γ=0 and δ=0, then spillover effects do not exist. The difference between the driving forces’ direct effects and their estimated coefficients is due to the feedback effects that travel through adjacent provinces and then back to the provinces themselves. The feedback effects consist of two parts: the value of the spatially lagged dependent variable (W*ln NOx) and the coefficients of the spatially lagged explanatory variables (population and energy intensity). Some prior EKC studies that applied the spatial econometric approaches either mistakenly reported the coefficient estimates as the direct and spillover effects, or avoided to report these effects in the SDM/SLM [18,20,36].

(13) $${\left[\begin{array}{ccc} \frac{\partial E(Y)}{\partial X_{1k}} & \ldots & \frac{\partial E(Y)}{\partial X_{nk}} \end{array}\right]}_{t}=\left[\begin{array}{ccc} \frac{\partial E(Y_{1})}{\partial X_{1k}} & \ldots & \frac{\partial E(Y_{1})}{\partial X_{nk}}\\ \vdots & \ddots & \vdots \\ \frac{\partial E(Y_{n})}{\partial X_{1k}} & \ldots & \frac{\partial E(Y_{n})}{\partial X_{nk}} \end{array}\right]={\left(I-\delta W\right)}^{-1}\left[\begin{array}{cccc} \beta_{k} & w_{12}\gamma_{k} & \cdots & w_{1n}\gamma_{k}\\ w_{21}\gamma_{k} & \beta_{k} & \cdots & w_{2n}\gamma_{k}\\ \vdots & \vdots & \ddots & \vdots \\ w_{n1}\gamma_{k} & w_{n2}\gamma_{k} & \cdots & \beta_{k} \end{array}\right]$$

(14) $$Y_{t}={\left(I-\;\delta W\right)}^{-1}\left(\mu +\eta \right)+{\left(I-\;\delta W\right)}^{-1}\left(X_{t}\beta +\gamma WX_{t}\right)+{\left(I-\;\delta W\right)}^{-1}\varepsilon_{t}$$

(15) $${\left(I-\;\delta W\right)}^{-1}=I+\delta W+\delta^{2}W^{2}+\delta^{3}W^{3}+\cdots$$

Another issue that has never been correctly discussed is the calculation of the turning points in the environmental Kuznets curve estimated by the SDM/SLM. Kang, Zhao, and Yang [26] applied a spatial econometric approach and found an inverse N-shaped CO_2_ EKC in China. However, they derived the turning points directly from the estimates of the GDP coefficients, which is invalid. The same problem occurred in Zhou, Ye, and Ge’s [19] study. In most situations, the EKC function is smooth, thus the limit points of the EKC function are the turning points. As for the SDM, its right-hand side contains the dependent variable, thus one needs to first derive the Equations (6)–(14) and then let the first-order derivative to be zero, so that the parameters for calculating EKC’s turning points can be obtained (here, we assume the EKC as a single variable function, since the EKC hypothesis solely focuses on the affluence’s impact). Thus, we argue that, when fitting the EKC by the SDM, one needs to apply the direct effects estimated through Equation (14), instead of using parameter estimates of the spatially lagged variable, to calculate the turning points.

Table 6 and Table 7 report the direct and spillover effects estimated according to Table 4 and Table 5 (fixed effects estimates). Model 1 (M1), Model 2 (M2), and Model 3 (M3) are respectively the GDP–nitrogen oxides/urbanization–nitrogen oxides models with cubic, quadratic, and linear terms of GDP/urbanization.

Turning our attention to the GDP–nitrogen oxides model results, the cubic, quadratic, and linear terms of the GDP’s coefficient (Table 4, M1) and direct effects (Table 6, M1) are statistically significant at a 5% level. Besides, the greater adjusted R^2^ and log likelihood (Table 4, M1) of the cubic model suggests that Model 1 (Table 6) fits the data better than Models 2 and 3 (Table 6). The significant effect estimates of energy intensity have the expected signs in Model 1. As we mentioned in Section 4.2.2, if we adopt the polynomial model, the SDM should not be simplified to the SLM or SEM. Therefore, the cubic form of the GDP–nitrogen oxides model is the appropriate specification for empirical analysis (the linear and quadratic models are inherently nested in the cubic model, therefor the cubic model should be adopted when parameters of linear and polynomial terms are significant at the same time).

**Figure 2:**
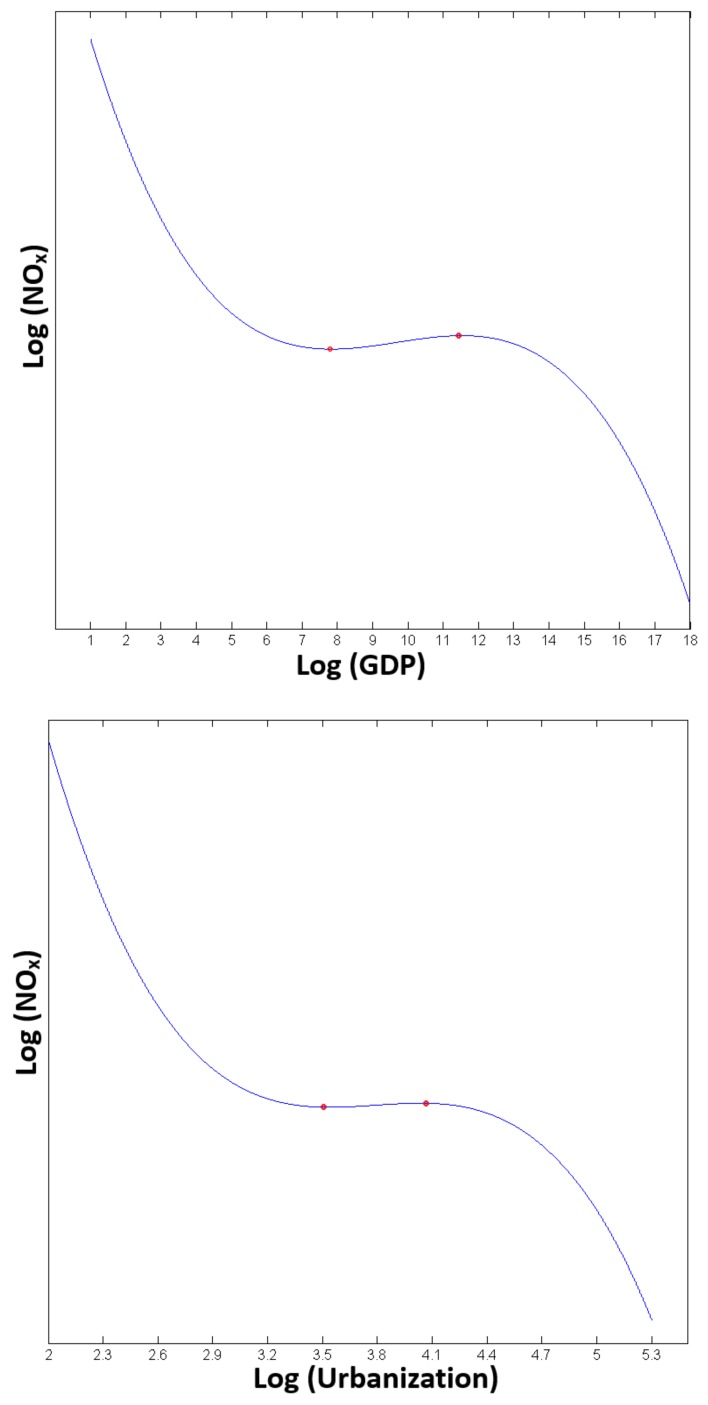
The partial fit of the GDP–NO_x_, Urbanization–NO_x_ emissions nexuses (logarithm transformed). Note: These two graphs aim to reveal the GDP–NO_x_ and Urbanization–NO_x_ relations, but not to predict NO_x_ emissions levels. Thus, the values on *Y*-axis are omitted. The turning points (marked with red dots) in the GDP–NO_x_ nexus are 7.8443 (2551 RMB) and 11.5403 (102,775 RMB), whereas in the Urbanization–NO_x_ nexus they are 3.5428 (35%) and 4.0373 (57%).

This finding shows that the estimated direct and spillover effects (elasticity) of energy intensity are highly significant at the 1% and 10% level respectively, and their signs are positive as expected. The effect of 1% growth in local energy intensity will lead to an increase in local nitrogen oxides emissions by 0.297%, other conditions being constant. LeSage and Pace [37] pointed out that the spillover effects are defined as the impact that a specific region exerts on all adjacent regions or vice versa. Thus, the impact of a 1% growth in local energy intensity will, on average, cause a 0.375% increase in nitrogen oxides emissions in neighboring provinces, all else being equal. On the other hand, both the direct and the spillover effects of the population are not significantly different from zero, which implies that a specific province’s population barely affects local and other provinces’ emissions. The highly significant linear, quadratic, and cubic terms of GDP per capita (Table 6, M1) point to an inversely N-shaped EKC for the nexus between NO_x_ emission and economic growth (Figure 2), which is consistent with the findings in prior China’s CO_2_ and SO_2_ EKC studies [19,20,26]. Moreover, two turning points of the inverse N-shaped trajectory are approximately 2551 Renminbi (RMB) and 102,775 RMB, respectively (these two turning points are estimated on the basis of the polynomial equation $\log NO_{x}=-0.052{\left(\log GDP\right)}^{3}+1.512{\left(\log GDP\right)}^{2}-14.122\log GDP$). Based on our sample, most of the economically developing provinces/cities (e.g., Guangxi, Xinjiang, and Qinghai provinces) are in the upward phase after the first turning point. There exists a general uptrend in nitrogen oxides emissions in such areas, and the personal incomes in the areas are between these two turning points. On the contrary, several developed cities with GDP per capita over 102,775 RMB (Beijing, Tianjin, and Shanghai) are experiencing a persisting decline in nitrogen oxides emissions. None of the observed per capita GDP is below 2551 RMB. The lowest one is 8237 RMB, in Guizhou province in 2010.

We will now turn to the Urbanization–nitrogen oxides model results. Similar to the GDP-nitrogen oxides model outcomes, all the polynomial terms of the urbanization’s coefficient (Table 5, M1) and direct effects (Table 7, M1) are statistically significant. Besides, the greater adjusted R^2^ and log likelihood (Table 5, M1) of the cubic model suggest that this model has the best explanatory power. Other than that, the energy intensity estimates remain positive, significant, and almost unchanged (0.240 and 0.355). Statistically, the population’s direct and spillover effects on emission are still not different from zero. Thus, the cubic Urbanization–nitrogen oxides model results are consistent with the cubic GDP–nitrogen oxides model results.

Because of the significant linear and polynomial terms of urbanization (Table 7, M1), we infer that there exists an inversely N-shaped EKC for the Urbanization–nitrogen oxides nexus (Figure 2), which is somewhat different from a prior study of China’s urbanization and industrial pollution [38]. This is probably because this prior study did not apply the EKC model as the theoretical foundation for its empirical analysis and applied different pollution indicators. Two turning points of the inverse N-shape trajectory are approximately 34.56% and 56.67%, respectively (these two turning points are estimated on the basis of the polynomial equation $\ln NO_{x}=-1.956{\left(\log URB\right)}^{3}+22.240{\left(\log URB\right)}^{2}-83.932\log UR\text{​}B$). In our sample, the urbanization levels of Beijing, Tianjin, Shanghai, and Guangdong and Jiangsu provinces are already over 56.67% at the beginning, and their local nitrogen oxides emissions indeed experienced downward trends as urbanization proceeded in the whole study period. Conversely, emissions in the rest of the provinces in the sample firstly experienced upward trends and then declined after approximately reaching 56.67%.

Unlike the population term, the autoregressive parameters of W log NO_x_ in the SDM (Table 4 and Table 5) are positive and statistically significant at the 1% level, which further testifies and demonstrates the spillover effects of nitrogen oxides emissions among the neighboring provinces. Specifically, a 1% increase and decrease of local nitrogen oxides emissions would lead to about a 0.3% corresponding variation in neighboring provinces and vice versa.

### 4.3. Discussion

The empirical findings in this research provide firm evidence of the spatial dependence of nitrogen oxides emissions and spillover effects of energy intensity at a provincial and municipal level in China. The highly significant global Moran’s I suggests HH and LL spatial clustering patterns of nitrogen oxides emissions. Overall, the eastern coastal and economically developed areas with higher levels of urbanization suffer more from nitrogen oxides emissions than the less developed regions with sparse population in urban areas. With the introduction of the spatial econometric analysis, this study empirically validates the economic development, urbanization progress, and energy intensity as the driving forces of nitrogen oxides emissions in China.

We tentatively put forward that the nitrogen oxides emissions spillover effects occur because of the imitation of neighbors’ economic and environmental policies. Some literature pointed out that such spatial patterns might originate from the government’s economically guided manipulation of environmental standards for attracting investment or for trade demand [39,40]. Thus, the implementation of environmental policies can be influenced by changes in neighboring countries and vice versa. Specifically, governors, officials, and bureaucrats might keep assessing their own policies by keeping an eye on the neighboring countries to simplify decision-making and shrink its costs. In this way, they can also legitimize their decisions, especially in the case that their policies might bring uncertain outcomes. As a result, the imitation of environmental policies by neighboring countries possibly leads to similar environmental standards and protection measures. In all these cases, externalities can transmit over the boundaries of countries/cities and contribute to the spatial effects on economic–environmental issues. The spillover in environmental policies among adjacent regions finds evidence also in the sociological literature [41,42].

In China, the central government assigns the national achievements of annual economic growth and pollution abatement at the provincial and county levels. The evaluation of such achievements is processed in each province and county. It is reasonable that competition arises among provinces for political performance in terms of economic development and emission abatement. If a local government implements rigorous controls on air pollution, the adjacent provinces may follow and implement similar ways to reduce emissions. On the contrary, if a local area still has a series of loose regulations on environmental protection and take the economic growth as its primary goal regardless of the air pollution, its neighbors would probably implement a similar strategy to catch up in terms of economic development. In this way, one can interpret the spillover effects as “demonstration effects” [36].

The GDP–nitrogen oxides model with the inclusion of the cubic term of GDP per capita has the best fit. In addition, the cubic-specified Urbanization–nitrogen oxides model is considered as the proper model. Based on these results, the enlightening and worthwhile finding of this study is that there exists an inversely N-shaped EKCs for both the GDP–nitrogen oxides nexus and the Urbanization–nitrogen oxides nexus. The former conclusion (inversely N-shaped EKC) is inconsistent with findings on nitrogen oxides emissions of a similar prior study. Brajer, Mead, and Xiao [12] asserted that there existed an inversely U-shaped EKC rather than an inversely N-shaped one for the nexus nitrogen oxides emissions–GDP per capita in China. We find two major reasons for the inconsistency: (a) our study applied the STIRPAT theory and control for the energy-related factors that significantly contribute to NO_x_ emission, while Brajer, Mead, and Xiao [12] did not; (b) we put forward the application of spatial panel data approaches to explore the relationship between nitrogen oxides emissions and economic development for the first time, whereas Brajer, Mead, and Xiao [12] did not control for the potential spatial autocorrelation among regional emissions even though their samples were also obtained from administrative areas that are usually spatially correlated. The combustion of fossil fuels from heating, power generation processes, and motor vehicles’ internal combustion engines is mainly responsible for the ambient NO_x_ emission. The introduction of the energy consumption term into the regression model is necessary and provides results that are more reasonable. This might explain the low fit (R^2^) in Brajer, Mead, and Xiao [12]. Moreover, Anselin and Rey [43] argued that this kind of spillover effect is essential, and incorrect omission would invalidate the inferences from a study, although it is difficult to practically designate the exact causes for spatial autocorrelation in the data examined. The existence of spatial relationships, after all, offers a potential explanation of instability in the parameters of the EKC. One of the speculations is that the behavior of neighboring countries would influence a country’s own action, which causes the spatial spillovers. Failure to control for spatially lagged variables may result in biased parameters of the EKC [22].

Our results are also similar to some recent findings on air pollution–economic growth/urbanization nexuses. After studying carbon emission and urbanization in 88 developing countries, Martínez-Zarzoso and Maruotti [29] found empirical evidence supporting the CO_2_ emission–urbanization EKC hypothesis. CO_2_ emission–urbanization EKC was also confirmed in Wang, Zhang, Kubota, Zhu, and Lu’s [25] study of The Organization for Economic Co-operation and Development (OECD) countries. In a dynamic spatial econometric panel analysis of carbon dioxide intensity, Zheng, Yu, Wang, and Deng [20] found evidence supporting the inversely N-shaped CO_2_ emission–income EKC hypothesis in China. Other than that, the CO_2_ emission–income EKC was also demonstrated to be inversely N-shaped in Kang, Zhao, and Yang’s [26] research on the relationship between CO_2_ emission and GDP per capita, as well as other potential driving forces in China. Wang et al. [44] and Zhou, Ye, and Ge [19] established inversely U- and inversely N-shaped EKCs, respectively, for the SO_2_ emission–GDP relation at China’s provincial level. On the other hand, the insignificant population impacts on pollutant emission suggested by our empirical results is inconsistent with the conclusions of these prior studies. This is probably because the CO_2_ emission is a comprehensive pollution indicator and it is broadly related to socioeconomic activities. Therefore, CO_2_ emission can reflect the direct influence of human factors. Different from CO_2_ emission, anthropogenic NO_x_ emissions mainly originate from the high-temperature combustion process of fossil fuels in industrial and automotive internal combustion engines, which can hardly be directly inferred from the total population size. This is because a province/region can have a large population but a very low individual vehicle occupancy volume. In this case, people are more dependent on public transport, which can improve energy efficiency in the transport sectors. Furthermore, if the public transport in such a city/region widely adopts environmental-friendly energy, the emissions level could decline. In short, a larger population generally means increased demand of transportation, but there is no absolute connection between NO_x_ emissions and total population. The energy efficiency and structure in the transport sectors are also key factors determining NO_x_ emissions levels.

What is the substantial mechanism behind the phase after the second turning point of the inverse N-shaped EKC in this analysis? There are several facts contributing to the impact on nitrogen oxides emissions related of economic development and urban sprawl. In an early stage, the economy developed speedily with a considerable consumption of energy, which caused a large air pollutants emission. As the social wealth accumulated to a certain point, pollution issues arouse wide media attention and broad concerns in the public. In order to deal with these pressures and reduce pollutants emission, the Chinese government has increased investments to decrease end-of-pipe emissions and tail gas pollution, and has promoted the use of alternative energy sources to fuels since the 11th Five-Year (2006–2010) Plan [45]. In the 12th Five-Year (2011–2015) plan, it was proposed that, in 2015, the total amount of NO_x_ emissions in the whole country should not have exceeded 20.462 million tons, which is 10% lower than the 22.736 million tons of 2010. To achieve this, the government has taken measures and launched a series of projects, such as the elimination of vehicles which did not reach the exhaust pollution control standard for motor vehicles, vehicle fuel replacement, low NO_x_ combustion retrofits in electric power and cement industries, etc. On the basis of the REN21 [46] report, China has a large amount of new energy sources, such as wind power, biofuels, solar power, and hydropower. As for the EKC of the urbanization effects, an early rapid urban sprawl leads to large amounts of building material consumption to upgrade and construct new infrastructures (e.g., drainage systems, road networks) which increase the energy use and the pollutant emissions in local areas. In addition, new immigrants to urban areas give rise to increasing demands for electricity, and China heavily relies on thermal power generation, which drives the rise of nitrogen oxides emissions. Once the urbanization level reaches a certain threshold, the higher population density in urban areas enables more efficient utilization of the public infrastructures, such as public transport, which may lower the energy consumption and consequently mitigate emission issues [47,48]. A relatively higher urbanization also comes with environmental improvements by economies of scale in terms of sanitation services and environmental protection [49]. Therefore, the “up-and-down” phase of the inverted N-shaped curve nexus of nitrogen oxides emissions and economic growth/urbanization is consistent with the EKC hypothesis. We need to remind the reader that, constrained by the sample interval, the mechanism of the first downward phase in the inverse N-shaped curve remains unclear.

## 5. Conclusions

This study, for the first time, examines the quantitative relationship between income/urbanization and nitrogen oxides emissions for China within the EKC hypothesis and the STIRPAT framework through a spatial panel data regression estimation. In comparison with conventional econometric approaches, spatial econometric techniques were never used before in the exploration of the nexus between nitrogen oxides emissions and income/urbanization. The parameters estimated by the spatial panel data model are more reliable than those obtained using conventional panel models because of the introduction of nitrogen oxides emissions’ spatial dependence on the characteristics of nearby provinces. Our results provide evidence suggesting that the relationships between income/urbanization and nitrogen oxides emissions shape inverse N curves that are different from the classical inverted U-shaped EKC curves at the current stage.

According to the findings of this analysis, the following policy suggestions are brought up for further mitigation of China’s nitrogen oxides emissions. In general, most provinces are in the second upward phase, while the rest few well-developed provinces are in the third downward phase in the inversely N-shaped trajectory. In other words, the rapid economic growth and urban sprawl with great nitrogen oxides emissions will not last for a long time in China, since the personal income in most provinces is either approaching or has already passed the second turning poins of per capita GDP and urbanization (102,775 RMB, 56.67%). Even though the society’s affluence accumulation could enable the government to invest more on pollution control and on the development of new types of energy to reduce pollutants emission, it is not wise to wait until the turning point is reached in the less developed provinces, because the environmental system cannot withhold pollution influences if pollution accumulation exceeds the threshold of total nitrogen oxides. Therefore, it is imperative for both the central and the local governments to implement policies and measures to limit the amount of local nitrogen oxides emissions instead of favoring the EKC hypothesis, so that economic development and urbanization will eventually benefit the environment. In addition, to deal with the adverse effects of the current rapid and continuous urban expansion, local governments should take steps to decelerate the sprawl of cities, encourage residents to take green commutes (e.g., promote the use of bicycles for short journeys), and invest more in technologies and facilities to treat air pollutants emission and improve energy consumption efficiency.

The significant spatial spillover effects of nitrogen oxides emissions suggest that policymakers, especially local governments, should not only focus on the local emission level but also consider the influence of the neighboring provinces. Meanwhile, China’s central government should make nationwide plans on emissions mitigation and define targeted nitrogen reduction goals for prefecture administrators, according to the different characteristics of each province. If necessary, the local governments should break administrative boundaries and associate for the enhancement of nitrogen oxides abatement and economic growth at the same time.

Although our findings in this study are illuminating, we tend to have a cautious attitude toward them because there are still some limitations related to them. First, given the fact that we applied a relatively short panel of the sample in the analysis, the time span of the data is limited and does not cover the first turning point of the inversely N-shaped KEC. In this regard, further research with longer panel data can enhance the knowledge of the fluctuation at the beginning of the inversely N-shaped curve. Second, in developed countries (e.g., Europe, North America, Japan), the average level of urbanization already reached 77.7% in 2011 [50]. This figure in China is just over 52%. Further studies, including data of the recent years, could provide more evidence for the relation between urbanization and emissions. Third, this research used the NO_x_ emissions as the pollutant index and made some conclusions on the relationship between development and NO_x_ emissions. However, these conclusions are not directly applicable to the relationship between development and NO_x_ concentration. This is another issue worth studying, because the concentration index is closely related to people’s health (morbidity and mortality) in daily life.

## Figures and Tables

**Table 1:** Definitions and descriptive statistics of the variables.

Variable	Definition	Mean	Std.Dev	Min	Max
log NO_X_	Nitrogen oxides emissions (ton)	13.292	0.703	11.294	14.404
log GDP	Real GDP per capita (RMB)	10.332	0.560	9.016	11.760
log URB	Percentage of urban population in the total population (%)	3.9793	0.221	3.521	4.495
log POP	Total Population	8.188	0.739	6.333	9.292
log EI	Energy intensity (Energy use per unit GDP, kg of coal equivalent/10000 GDP)	7.052	0.486	6.084	8.260

Note: The real GDP per capita was measured by the 2003 constant price; RMB refers to Renminbi, the official currency of the People’s Republic of China; log NO_x_, log POP, and log EI are the proxies of environmental impact, population size, and technical impacts in Equations (3)–(5); log GDP and log URB (urbanization) are the proxies of affluence in Equations (3)–(5).

**Table 2:** NO_x_ emissions’ Global Moran’s I statistics.

	2010	2011	2012	2013	2014	2015
Moran’s I	0.212	0.190	0.186	0.173	0.173	0.182
Z-Score	2.327	2.121	2.080	1.959	1.963	2.052
*p*-value	0.020	0.034	0.038	0.050	0.050	0.040

Note: For consistency with the regression analysis, the spatial weight matrix W for the Moran’s *I* test was also row-normalized.

**Table 3:** Parameter estimates of the non-spatial panel model.

Dependent Variable: logNOx	Per Capita GDP as the Index of Affluence			Urbanization as the Index of Affluence		
	M1	M2	M3	M1	M2	M3
log A	−15.272 ***	1.972 ***	0.745 ***	−17.495	2.789	0.448 ***
	(−3.041)	(5.108)	(6.453)	(−0.535)	(1.513)	(3.109)
(log A)^2^	1.601 ***	−0.074 ***		4.943	−0.318	
	(3.288)	(−3.322)		(0.584)	(−1.274)	
(log A)^3^	−0.054 ***			−0.454		
	(−3.443)			(−0.622)		
Log POP	0.271	−0.284	−0.988 ***	−0.402	−0.444 *	−0.434 *
	(0.810)	(−0.940)	(−4.460)	(−1.556)	(−1.785)	(−1.741)
Log EI	0.393 ***	0.444 ***	0.503 ***	0.471 ***	0.480 ***	0.481 ***
	(7.291)	(8.309)	(9.706)	(8.157)	(8.579)	(8.598)
LM test no spatial lag	6.3766 **	7.1485 ***	10.4617 ***	15.8132 ***	15.3210 ***	16.8318 ***
robust LM test no spatial lag	0.0136	0.2819	0.0079	2.2739	2.7814 *	1.1501
LM test no spatial error	8.0278 ***	7.7327 ***	13.1395 ***	13.5398 ***	12.5722 ***	15.9735 ***
robust LM test no spatial error	1.6647	0.8661	2.6858	0.0005	0.0326	0.2917
LR-test spatial fixed effects	749.7847 ***	740.0405 ***	747.0445 ***	713.5834 ***	715.3909 ***	732.1524 ***
LR-test time fixed effects	186.7664 ***	175.4269 ***	179.2426 ***	154.7254 ***	155.5018 ***	157.1568 ***
N	180	180	180	180	180	180
Rbar-squared	0.4877	0.4561	0.4253	0.3268	0.3291	0.3268

Note: Numbers in the parentheses are t-stat; * *p* < 0.1; ** *p* < 0.05; *** *p* < 0.01. M1, M2, and M3 refer to the models corresponding to Equations (3)–(5), respectively; log A: logarithm of affluence; log POP: logarithm of total population; log EI: logarithm of energy intensity; LM test: Lagrange Multiplier test; LR-test: likelihood ratio test

**Table 4:** Parameter estimates of the spatial panel model (GDP as the indicator of affluence).

Dependent Variable: logNOx	Fixed Effects Estimates			Random Effects Estimates		
	M1	M2	M3	M1	M2	M3
logGDP	−14.886 ***	2.146 ***	0.715 ***	−16.737 ***	2.513 ***	0.306 ***
	(−2.796)	(4.083)	(5.002)	(−3.395)	(5.583)	(2.983)
(logGDP)^2^	1.587 ***	−0.088 ***		1.761 ***	−0.108 ***	
	(3.047)	(−3.151)		(3.678)	(−5.098)	
(logGDP)^3^	−0.055 ***			−0.060 ***		
	(−3.225)			(−3.894)		
logPOP	0.257	−0.197	−0.891 **	0.787 ***	0.768 ***	0.773 ***
	(0.592)	(−0.466)	(−2.532)	(9.760)	(9.596)	(9.721)
log EI	0.272 ***	0.345 ***	0.455 ***	0.320 ***	0.355 ***	0.461***
	(3.541)	(4.523)	(6.403)	(4.834)	(5.206)	(6.376)
WlogGDP	13.293	0.667	−0.402 *	2.723	−0.199	−0.045
	(1.064)	(0.784)	(−1.752)	(0.252)	(−0.276)	(−0.265)
(WlogGDP)^2^	−1.354	−0.088 *		−0.320	−0.006	
	(−1.110)	(−1.734)		(-0.304)	(-0.161)	
(WlogGDP)^3^	0.042			0.011		
	(1.069)			(0.321)		
WlogPOP	0.441	0.644	0.211	−0.320 *	−0.394 **	−0.627 ***
	(0.613)	(0.923)	(0.343)	(−1.945)	(−2.411)	(−3.978)
Wlog EI	0.144	0.074	−0.080	0.079	0.021	−0.149
	(1.006)	(0.501)	(−0.547)	(0.587)	(0.150)	(−1.003)
W*log NOx	0.376 ***	0.350 ***	0.426 ***	0.313 ***	0.318 ***	0.425 ***
	(4.536)	(4.120)	(5.291)	(3.731)	(3.810)	(5.614)
teta				0.043 ***	0.045 ***	0.051 ***
				(5.481)	(5.481)	(5.482)
Hausman				26.9213 ***	40.1047 ***	75.0638 ***
N	180	180	180	180	180	180
Rbar−squared	0.5397	0.5163	0.4263	0.7208	0.7131	0.7429
Wald_spatial_lag	16.0228 ***	13.3801 ***	3.3914	15.3446 ***	15.3468 ***	17.7147 ***
LR_spatial_lag	16.9816 ***	15.3136 ***	2.7463	13.1342 ***	13.5632 ***	15.6328 ***
Wald_spatial_error	12.5273 **	11.9753 **	1.2424	8.5388	8.9657 *	5.5823
LR_spatial_error	15.2257 ***	14.8212 ***	1.3204	12.8486 ***	13.0801 ***	9.6508 **

Note: Numbers in the parentheses are t-stat; * *p* < 0.1; ** *p* < 0.05; *** *p* < 0.01. M1, M2, and M3 refers to the models corresponding to Equations (3)–(5), respectively.

**Table 5:** Parameter estimates of the spatial panel model (urbanization as the indicator of affluence).

Dependent Variable: logNOx	Spatial Fixed Effects			Spatial Random Effects		
	M1	M2	M3	M1	M2	M3
logURB	−86.326 **	1.505	0.523 ***	−71.725 **	1.868	0.589 ***
	(−2.269)	(0.799)	(2.687)	(−2.475)	(1.076)	(3.446)
(logURB)^2^	22.580 **	−0.154		18.711 **	−0.172	
	(2.278)	(−0.609)		(2.508)	(−0.751)	
(logURB)^3^	−1.958 **			−1.610 **		
	(−2.276)			(−2.520)		
logPOP	−0.333	−0.395	0.104	0.737 ***	0.766 ***	0.817 ***
	(−1.022)	(−1.189)	(0.314)	(8.598)	(9.083)	(10.402)
log EI	0.215 ***	0.277 ***	0.374 ***	0.311 ***	0.379 ***	0.425 ***
	(2.792)	(3.705)	(4.978)	(4.222)	(5.132)	(5.939)
WlogURB	44.950	20.596 ***	−0.454	119.975 *	5.661	−0.319
	(0.530)	(4.604)	(−1.522)	(1.944)	(1.541)	(−1.166)
(WlogURB)^2^	−8.769	−2.881 ***		−30.056 *	−0.813 *	
	(−0.396)	(−4.707)		(−1.891)	(−1.643)	
(WlogURB)^3^	0.464			2.485 *		
	(0.241)			(1.821)		
WlogPOP	−0.758	−0.889	−0.864	−0.541 ***	−0.565 ***	−0.594 ***
	(−1.351)	(−1.545)	(−1.461)	(−3.346)	(−3.504)	(−3.811)
Wlog EI	0.173	0.145	0.020	−0.017	−0.079	−0.080
	(1.196)	(0.974)	(0.131)	(−0.120)	(−0.538)	(−0.541)
W*log NOx	0.343 ***	0.328 ***	0.469 ***	0.444 ***	0.410 ***	0.430 ***
	(4.068)	(3.827)	(6.094)	(5.975)	(5.318)	(5.695)
teta				0.044 ***	0.047 ***	0.050 ***
				(5.481)	(5.481)	(5.482)
N	180.000	180.000	180.000	180.000	180.000	180.000
Rbar-squared	0.512	0.485	0.337	0.632	0.657	0.719
Hausman				26.426 ***	32.269 ***	22.014 ***
Wald_spatial_lag	35.5964 ***	26.9514 ***	4.1150	27.9652 ***	21.0943 ***	18.6102 ***
LR_spatial_lag	39.2325 ***	30.8808 ***	4.5914	24.5783 ***	19.0932 ***	16.1401 ***
Wald_spatial_error	35.2548 ***	28.3682 ***	5.1504	13.3523 **	9.2156 *	5.7002
LR_spatial_error	39.5535 ***	32.8361 ***	6.1206	16.9972 ***	12.0617 **	8.6987 **

Note: Numbers in the parentheses are t-stat; * *p* < 0.1; ** *p* < 0.05; *** *p* < 0.01. M1, M2, and M3 refer to the models corresponding to Equations (3)–(5), respectively.

**Table 6:** Direct and spillover effects estimation (gross domestic product as the indicator of affluence).

	M1		M2		M3	
	Direct	Spillover	Direct	Spillover	Direct	Spillover
logGDP	−14.122 **	11.353	2.281 ***	2.104 *	0.697 ***	−0.155
	(−2.530)	(0.603)	(4.629)	(1.885)	(4.988)	(−0.493)
(logGDP)^2^	1.512 ***	−1.117	−0.100 ***	−0.174 **		
	(2.772)	(−0.610)	(−3.636)	(−2.386)		
(logGDP)^3^	−0.052 ***	0.032				
	(−2.965)	(0.540)				
logPOP	0.296	0.856	−0.123	0.822	−0.901 ***	−0.272
	(0.702)	(0.858)	(−0.315)	(0.935)	(−2.865)	(−0.329)
log EI	0.297 ***	0.375 *	0.359 ***	0.303	0.468 ***	0.187
	(4.322)	(1.939)	(5.008)	(1.635)	(7.247)	(0.908)

Note: Numbers in the parentheses are t-stat; * *p* < 0.1; ** *p* < 0.05; *** *p* < 0.01; the direct and spillover effects of linear, square, and cubic terms of log GDP are practically meaningless; M1, M2, and M3 refer to the models corresponding to Equations (3)–(5), respectively.

**Table 7:** Direct and spillover effects estimation (Urbanization as the indicator of affluence).

	M1		M2		M3	
	Direct	Spillover	Direct	Spillover	Direct	Spillover
logURBEN	−83.932 *	22.233	3.404 *	29.880 ***	0.501 ***	−0.377
	(−1.904)	(0.164)	(1.775)	(4.319)	(2.749)	(−0.850)
(logURBEN)^2^	22.240 *	−1.504	−0.416	−4.156 ***		
	(1.930)	(−0.042)	(−1.603)	(−4.353)		
(logURBEN)^3^	−1.956 *	−0.297				
	(−1.948)	(−0.096)				
logPOP	−0.415	−1.268	−0.464	−1.477 *	−0.019	−1.404
	(−1.364)	(−1.680)	(−1.490)	(−1.979)	(−0.062)	(−1.524)
log EI	0.240 ***	0.355 *	0.301 ***	0.333 *	0.403 ***	0.349
	(3.223)	(1.919)	(4.207)	(1.744)	(5.930)	(1.478)

Note: Numbers in the parentheses are t-stat; * *p* < 0.1; ** *p* < 0.05; *** *p* < 0.01; the direct and spillover effects of linear, square, and cubic terms of log URB are practically meaningless. M1, M2, and M3 refer to the models corresponding to Equations (3)–(5), respectively.
